# The case report of *Mycobacterium arupense* wound infection in diabetes mellitus patients; the first report and literature review

**DOI:** 10.1099/acmi.0.000106

**Published:** 2020-02-17

**Authors:** Sepehr Navid, Bahar Sadegh-Ehdaei, Mehdi Shabani, Melika Hasani, Arezoo Mirzaei, Kiarash Ghazvini, Masoud Youssefi, Masoud Keikha

**Affiliations:** ^1^​ Department of Microbiology, School of Medicine, Isfahan Medical University, Isfahan, Iran; ^2^​ M.Sc. of Molecular Genetics, Sana Institue of Higher Education, Sari, Iran; ^3^​ Antimicrobial Resistance Research Center, Mashhad University of Medical Sciences, Mashhad, Iran; ^4^​ Department of Microbiology and Virology, Faculty of Medicine, Mashhad University of Medical Sciences, Mashhad, Iran

**Keywords:** *Mycobacterium arupense*, diabetes mellitus, wound infection, 16S rRNA

## Abstract

*
Mycobacterium arupense* is among the opportunist pathogens of atypical mycobacteria emergence (atypical mycobacteria) that is one of the isolated and reported environmental and clinical specimens. Numerous cases of osteo-articular infections of this bacterium are reported nowadays, while the pulmonary infection is rare. We identified *Mycobacterium arupense* in non-healing wound infection of an elderly woman with history of diabetes mellitus. She has negative tests for HIV, HBV and HCV, but was positive for HTLV-1. The patient was referred according to mild-fever, non-healing, destructive, and swelled lesion on her left foot. The mycobacterial wounds infection was suspected due to her non-conclusive previous treatment. The pathology, acid-fast staining, conventional and 16S rRNA sequencing confirmed the micro-organism to be *M. arupense *. Finally, the patient recovered following two-week consumption of clarithromycin, ethambutol and rifabutin. The results of this study provide evidence on the potential pathogenicity, clinical outcomes and treatment of infections caused by this bacterium.

## Introduction

Non-tuberculosis mycobacteria (NTM) are a group of ‘Mycobacteria’ that live in environmental resources such as saprophytes and that enter their body through inhalation and traumatic inclusion, causing the mycobacterosis infection [1, 2]. The incidence rate of NTM infections is increasing nowadays [3, 4]. The improved diagnostic methods, especially the molecular diagnostic methods, and the increased number of immune-disorders have increased the rate of NTM infections [5].

*Mycobacterium arupense* was first isolated from a tendon sample in 2006 and identified by Cloud *et al*. [6]. *M. arupense* is part of the *M. terrae* complex and is very similar to *M. nonchromogenicum* [6, 7]. Identification of this type of clinical sample is quite difficult due to the similarity of phenotypic tests with the members of *M. terrae* complex. However, the 16S rRNA gene in *M. arupense* is as a signature sequence and the sequencing of housekeeping genes, especially 16S rRNA, is able to correctly identify this species [6, 8]. According to the American Thoracic Society (ATS) guidelines, it is recommended that the NTM isolates isolated from clinical specimens should be identified to the species level for the final diagnosis, accurate identification, patient management, appropriate treatment and epidemiological goals [9].

There are numerous reports about the *M. arupense* isolation from respiratory, tenosynovitis, osteoarticular, osteomyelitis and disseminated infections [7, 8, 10–12]. The present study was the first case report of cutaneous infection by *M. arupense* in a HTLV-1-infected diabetic patient (HTLV-1 infected).

## Case presentation

A 51-year-old woman referred to Al-Zahra Hospital in Isfahan (Isfahan, Iran) in June 2018 due to non-healing foot ulcers in her left foot. She was a housewife living in a rural area near Faridan, Iran, working on farms and having a previous experience of foot ulcers. However, she stated that her recent foot ulcer had not healed in the last 1.5 months. The patient had a history of diabetes mellitus (since 2011). On initial examination, the patient had a mild fever (37.8 °C), and a swollen, necrotic ulcer was evident on the toes, and according to the patient, the lesions were not very painful. Sampling was done from the ulcer. Based on the microbiology lab reports, *Staphylococcus aureus* and *Klebsiella pneumonia* were isolated and penicillin, doxycycline, imipenem and bandage with Betadine were prescribed for the patient.

**Fig. 1.**
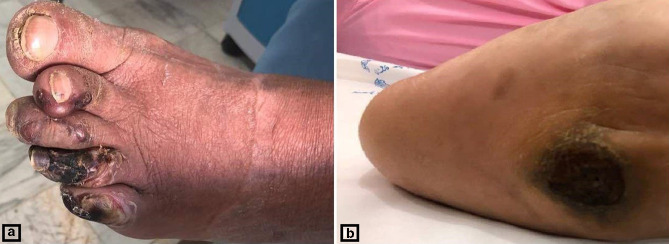
Wound lesion on the right foot of the patient. (A) lesions on the toes, (B) extended lesion on her sole.

The patient returned again about a month later due to failure to respond to treatment, although she reported painful scarring and pale discharge; lesion depth was 1.5 cm and also extended to her sole (Fig. 1). The patient had a temperature of 38.2 °C and according to MRI abdominal cavity and chest X-ray, she had no signs of inflammation in her lungs and internal organs.

The Fasting Blood Sugar (FBS) level was 126 mg dl^–1^; also Count Blood Cell (CBC) included: WBC: 11 500 μl^–1^, RBC: 4500 μl^–1^, Hb: 15.3 g dl^–1^, HCT: 44 % and transferases hepatic abnormalities were slightly elevated; patient CRP and ESR were also evaluated at 61 mg l^–1^ and 56 mm h^–1^, respectively. The patient had negative signs of HIV, HCV, and HBV, but the signs of HTLV-1 was positive (the titer of HTLV-1 virus in the patient blood was 12.8 copies per 100 cells).

**Fig. 2.**
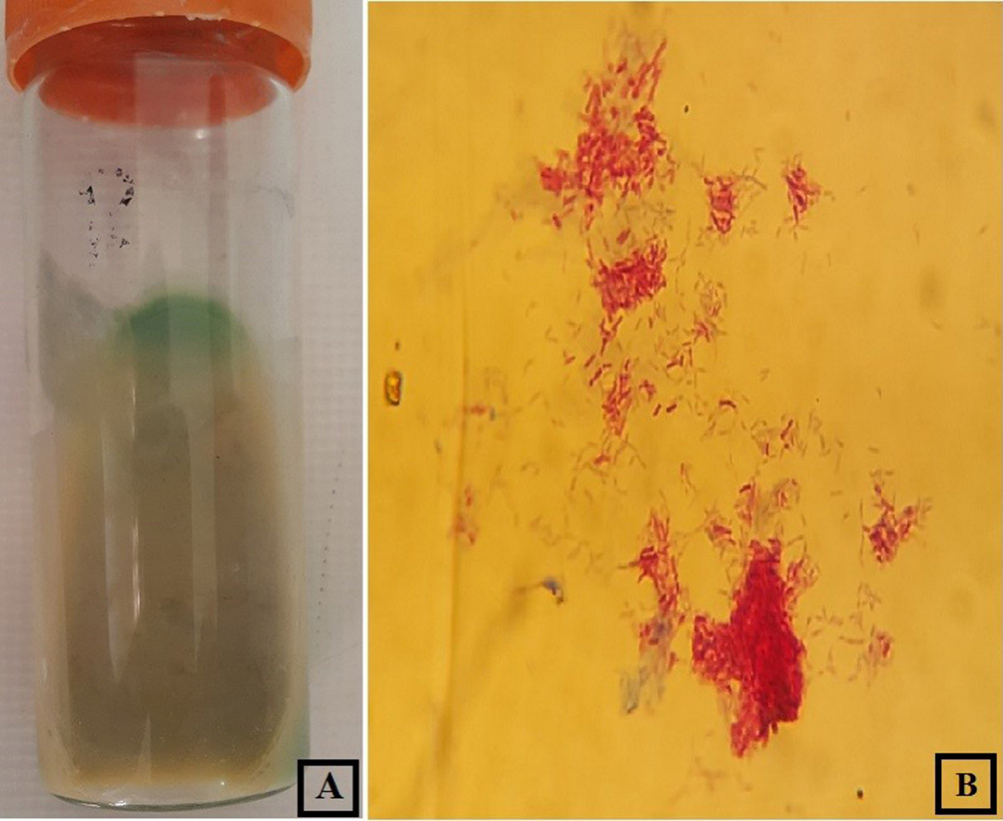
Colony morphology (a) and acid-fast staining (b) of *Mycobacterium arupense *.

The pathology evaluation revealed the presence of granuloma. Blood culture of the patient was negative, and wound exudate samples were examined using Gram-staining and Ziehl-Neelsen staining. Acid-fast bacilli were confirmed in the wound exudate, and subsequently, wound exudate samples were cultured on blood agar and Lowenstein Jensen slant. Two weeks later small colonies appeared on LJ enriched in Sauton's broth (Fig. 2).

**Fig. 3.**
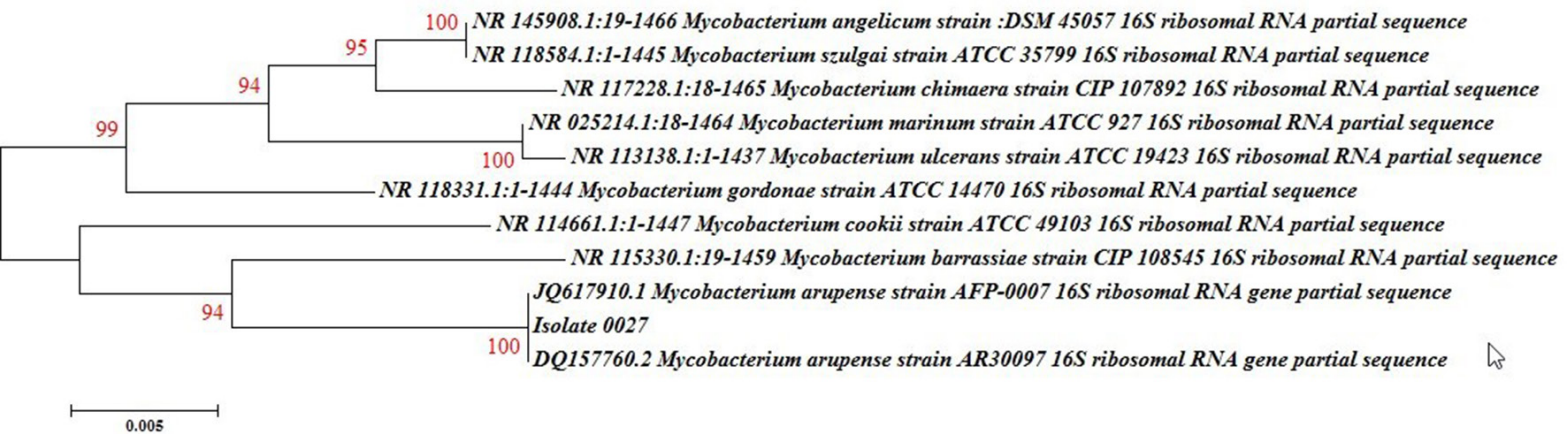
16S rRNA sequence-based phylogenetic tree of our isolate and closely related mycobacterial species which are conducted using mega with the neighbour-joining method and K2P distance.

The considered isolate was identified as rapidly growing mycobacteria (RGM) due to the growth rate (<7 days), lack of pigment production, negative results for niacin and nitrate reductase as well as urease and heat stable catalase production (68 °C). Molecular analysis was performed to identify to the species level. Simple boiling method was used to extract the DNA, the amplification of nearly full-length of 16S rRNA was performed by primers pA: 5′ AG-AGA GTTTGATCCTGGCTCAG-3′ and pI: 5′-TGCACACAGGCCACAAGGGA-3′ according to Rogall *et al*., and the sequence of PCR product was analysed [13]. NTM spp. can be differentiated by high-precision via nucleotide sequence of the hypervariable regions A (125-270) and B (408-503) of the 16S rRNA. In addition, the nucleotide sequence of rRNA gene of a short helix region is between the 451–482 positions that is a signature for RGM [14]. Based on the results by Blast, it was found that the partial sequence 16S rRNA of the considered isolation was 100 % similar to *Mycobacterium arupense* (DQ157760). A phylognic-relationship analysis based on closely related mycobacterial species also identified the isolate as *M. arupense* as accession number: MN865166 (Fig. 3).

Drug susceptibility test (DST) was performed according to CLSI M24-A2 recommendations by the broth micro-dilution method. Based on the DST results, the considered isolate was sensitive to clarithromycin, ethambutol, and rifabutin antibiotics and resistant to isoniazid, rifampicin, amikacin, moxifloxacin, ciprofloxacin and linezolid. Finally, the treatment was done with purulent drainage, initiation with clarithromycin, ethambutol and rifabutin together with once applying of interferon alpha to reduce the proviral load HTLV-1. After two weeks of antibiotictherapy the foot wound infection of the patient recovered and the patient was discharged with personal consent.

## Discussion

There are various evidences of isolation of *M. arupense* from environmental samples nowadays, such as surface water, soil, fish tanks, animal urine, and duck houses [15, 16]. Despite the widespread presence of this bacterium, there have been limited reports of human infections with *M. arupense* [16, 17]. The present study was the first case report of a diabetic person foot ulcer infection by *M. arupense *. Due to the limitations of *M. arupense *'s clinical reports, there is no standard guideline for the treatment of infections of this bacterium [17]. According to the review of the literature, *M. arupense* infections are more common in people with immune-disorders (Table 1).

**Table 1.** The summaries of clinical case reports of infection with *M. arupense
*

The authors	Cases	Risk factors	Diagnostic method	Treatment	Duration	Clinical outcome	Location	Year	Ref
Lopez *et al*.	Tenosynovitis	Multiple immunomodulatory drugs	Culture	Clarithromycin (500 mg 2×/d) Ethambutol (1,200 mg/d) Rifabutin (300 mg/d)	12	improved	USA	2016	[7]
Tsai *et al*.	Tenosynovitis	Diabetes mellitus	Sequencing of 16S rRNA	Clarithromycin (500 mg every 12 h), moxifloxacin (400 mg daily), rifabutin (300 mg daily), ciprofloxacin (400 mg every 12 h), and ethambutol (1000 mg daily)	6	improved	Taiwan	2008	[25]
Slany *et al*.	Pulmonary (3 cases)	Diabetes mellitus (case 1) Chronic gastritis (case 2) – (case 3)	Culture and 16S rRNA sequencing	Tuberculosis therapy	1–3 months	improved	Czech Republic	2010	[16]
Lee *et al*.	Tenosynovitis	Puncture injury	16S rRNA sequencing	Clarithromycin, ethambutol, and rifampin	nr	nr	South Korea	2014	[26]
Heidarieh *et al*.	Pulmonary (case 1) Disseminated (case 2)	HIV-infected	Culture and 16S rRNA sequencing	Clarithromycin, ethambutol, and rifampin	nr	nr	Iran	2013	[8]
Seidl *et al*.	Osteoarticular	Traumatic knee arthrotomy	Culture	Azithromycin, rifampin, and ethambutol	24	improved	Colorado	2014	[10]
Beam *et al*.	Flexor Tenosynovitis	Hypertension and hyperlipidemia	16S rRNA sequencing	Rifabutin, ethambutol, and clarithromycin and surgical drainage	6	improved	USA	2014	[27]
Neonakis *et al*.	Pulmonary	Large deficiency of the mitral valve and hypertension	Hsp65-RFLP	Rifabutin, ethambutol, and clarithromycin	nr	nr	Greece	2009	[20]
Legouta *et al*.	Osteomyelitis	Immunocompetent	Culture and hsp65 and 16S rRNA sequencing	Ciprofloxacin, ethambutol, amikacin	12 month	improved	France	2012	[28]
Senda et al.	Tenosynovitis	Arterial hypertension	DNA–DNA hybridization	Surgery and Rifampin, ethambutol	14	improved	Japan	2015	[29]
Zhou *et al*.	Pleural effusion	Immunocompetent	16S rRNA sequencing	Capreomycin and moxifloxacin (No NTM treatment)	nr	improved	China	2018	[21]

Currently, human infections caused by *M. arupense* are divided into two categories: pulmonary and extra-pulmonary infections. Based on the existing reports, most of these people have trauma, HIV, or corticosteroids use [17, 18]. Regarding the limited available information, it is not possible to fully understand the clinical significance, clinical outcome and duration of treatment of this bacterium [17].

However, surgical and antimicrobial therapy methods are commonly used for tenosynovitis and osteo-articular infections, whereas disseminated infections initiated with rifabutin, clatrithomycin and ethambutol have had satisfactory results. Furthermore, treatment regarding the pulmonary infection is based on ethambutol, clatrithomycin, rifabutin and drug susceptibility test; TMP-SXT results were also varied (Table 1).

According to the review of the literatures, the duration of treatment for *M. arupense* infections varies between 6 and 24 months, depending on the type of infection and the involved tissue, and includes a combination of surgery and antibiotic therapy. No signs of relapse or re-infection were reported after the treatment (Table 1). Also, most reports have shown that *M. arupense* clinical isolates are sensitive to clarithromycin, rifabutin, ethambutol and rarely to quinolones (Table 1).

In a study on *M. arupense* infections in cancer patients, Hamal *et al*. observed that the clinical outcome showed no significant difference between the treated *M. arupense* infected cancer patients treated and the untreated group; there were no reports of relapse or death from *M. arupense* [18]. Vasireddy *et al*. reported in their studies 10 strains of *M. arupense* tissue specimens that most of these patients had experience of trauma or using corticosteroids [19]. Currently, *M. arupense* is considered an emergent pathogen for osteoarticular infection. However, the role of this bacterium as a respiratory system pathogen is still unknown [20–22]. Pulmonary infections caused by *M. arupense* have been so far observed only in immune-deficiency patients (Table 1).

In this study, we present the first report of an unusual cutaneous infection caused by *M. arupense* in Iran. Patient's immune system of the present study was weakened by infection with HTLV-1 and diabetes mellitus, and according to the evidence, this bacterium is more likely to cause opportunistic infections in the individuals with immune system deficiency.

Identification of *M. arupense* is very important in TB-indemic regions, especially in Iran. Due to the slow growth of mycobacterium tuberculosis in the developing countries such as Iran, the considered patient affected by TB is reported only by observing acid-fast bacilli in smears of clinical specimens and considering a TB-endemic area [23]. This report demonstrates the importance of culture and identification to the species level of mycobacteria for appropriate diagnosis and treatment [6]. Based on the available evidence, two reports of infection with *M. arupense* in Iran have been reported indicating circulation of this bacterium in this geographical area [8]. The study was also the first report of cutaneous infection by this bacterium, indicating the potential pathogenicity of this microorganism.

Finally, the importance of molecular methods in identifying NTM spp. should be mentioned. Conventional and culture methods are expensive due to the slow growing nature of mycobacteria, and are not quite appropriate due to their inconclusive state, whereas molecular methods, especially 16S rRNA sequencing, are able to identify NTM species in high accuracy, in addition to being non-expensive and fast [24].
